# Post-Mortem Detection of SARS-CoV-2 RNA in Long-Buried Lung Samples

**DOI:** 10.3390/diagnostics11071158

**Published:** 2021-06-24

**Authors:** Nicolò Musso, Luca Falzone, Stefano Stracquadanio, Dafne Bongiorno, Monica Salerno, Massimiliano Esposito, Francesco Sessa, Massimo Libra, Stefania Stefani, Cristoforo Pomara

**Affiliations:** 1 Laboratory of Molecular and Resistant Antibiotic Medical Microbiology (MMAR), Department of Biomedical and Biotechnological Sciences (BIOMETEC), University of Catania, 95123 Catania, Italy; nmusso@unict.it (N.M.); s.stracquadanio@unict.it (S.S.); d.bongiorno@unict.it (D.B.); 2 Laboratory of Experimental Oncology, Department of Biomedical and Biotechnological Sciences (BIOMETEC), University of Catania, 95123 Catania, Italy; lucafk92@hotmail.it (L.F.); m.libra@unict.it (M.L.); 3 Department of Medical and Surgical Sciences and Advanced Technologies “G.F. Ingrassia”, Institute of Legal Medicine, University of Catania, 95123 Catania, Italy; monica.salerno@unict.it (M.S.); massimiliano.esposito91@gmail.com (M.E.); cristoforo.pomara@unict.it (C.P.); 4 Department of Clinical and Experimental Medicine, University of Foggia, 71122 Foggia, Italy; francesco.sessa@unifg.it

**Keywords:** COVID-19, autopsy, SARS-CoV-2, post-mortem swab, viral RNA

## Abstract

The Coronavirus Disease 19 (COVID-19) pandemic has caused an unexpected death toll worldwide. Even though several guidelines for the management of infectious corpses have been proposed, the limited number of post-mortem analyses during the pandemic has led to inaccuracies in the counting of COVID-19 deaths and contributed to a lack of important information about the pathophysiology of the SARS-CoV-2 infection. Due to the impossibility of carrying out autopsies on all corpses, the scientific community has raised the question of whether confirmatory analyses could be performed on exhumed bodies after a long period of burial to assess the presence of SARS-CoV-2 RNA. Post-mortem lung samples were collected from 16 patients who died from COVID-19 infection and were buried for a long period of time. A custom RNA extraction protocol was developed to enhance extraction of viral RNA from degraded samples and highly sensitive molecular methods, including RT-qPCR and droplet digital PCR (ddPCR), were used to detect the presence of SARS-CoV-2 RNA. The custom extraction protocol developed allowed us to extract total RNA effectively from all lung samples collected. SARS-CoV-2 viral RNA was effectively detected in all samples by both RT-qPCR and ddPCR, regardless of the length of burial. ddPCR results confirmed the persistence of the virus in this anatomical niche and revealed high viral loads in some lung samples, suggesting active infection at the time of death. To the best of our knowledge, this is the first study to demonstrate the persistence of SARS-CoV-2 viral RNA in the lung even after a long post-mortem interval (up to 78 days). The extraction protocol herein described, and the highly sensitive molecular analyses performed, could represent the standard procedures for SARS-CoV-2 detection in degraded lung specimens. Finally, the innovative results obtained encourage post-mortem confirmatory analyses even after a long post-mortem interval.

## 1. Introduction

The Coronavirus Disease 19 (COVID-19) pandemic has urged the scientific community to join forces in order to acquire the best knowledge of SARS-CoV-2 biology and clinical impact [1]. In Italy, probably due to the high average age of the population, the pandemic event caused an unexpected number of deaths in hospitals and long-term care facilities. Specific guidelines have been since issued for the management of suspected COVID-19 deaths in health care facilities and mortuaries [2], based on the World Health Organization recommendations [3]; these state that the leakage of body fluids should be contained and corpses should be treated with chlorine before being wrapped in plastic bags and buried. Unfortunately, the high death toll and the risks for the physicians caused post-mortem analyses to be often performed too rapidly, resulting in a lack of a well-defined pathophysiology of death among patients who died following COVID-19 infection, as well as overlooking potential key information about the real mechanisms underlying these deaths [4,5], leading to probable errors in the estimation of COVID-19 mortality.

One question that should be answered to unveil the actual amount of true COVID-19 deaths regards viral survival and detectability in body parts after corpse processing and burial, even if analyses are performed after a long post-mortem interval. Moreover, corpses and their parts, especially infected lungs, may be other possible niches where the virus can continue to live undisturbed [4].

To date, only a few papers [6,7] report on molecular analyses of swabs performed during clinical autopsies with the aim of developing guidelines to minimize the risk of infection for coroners, but there is a lack of information about virus integrity in buried bodies.

Furthermore, a standard protocol for the extraction of viral RNA from body parts still does not exist and RT-qPCR remains the gold standard to evaluate the presence of SARS-CoV-2 [8,9]. However, this method is affected by the quality of nucleic acids. Besides, DNA degradation may give false negative results, resulting in a bias in the evaluation of the COVID-19-related deaths.

In this context, the aim of the present study was to determine detectability of SARS-CoV-2 viral RNA in long-buried corpses of patients who died with a diagnosis of COVID-19 infection by using both quantitative Real-Time PCR (RT-qPCR) assay and droplet digital PCR (ddPCR).

## 2. Materials and Methods

### 2.1. Patients, Autopsies and Sample Collection

The present study was performed during the first wave of COVID-19 pandemic in Italy. Despite the recommendation of the Italian Government to significantly limit clinical and forensic investigation on potentially infected corpses, we included a case series of 16 consecutive COVID-19 patients who died in an Italian long-term care facility from April 2020 to May 2020 and two COVID-19 patients who died at the San Marco Hospital in Catania (Italy) in September 2020. For the first 16 COVID-19 patients, autopsies were performed in June 2020, approximately two months after death and normal burial, whereas the other two patients were autopsied within 48 h of death and were used as positive controls for the detection of SARS-CoV-2 RNA. Autopsies were conducted following international guidelines [2,3,10]. All autopsies were conducted according to the Letulle method [11]. Prior to fixation, three tissue fragments from the right lung and two from the left lung were collected and immediately transferred to sterile vials containing RNA Later (Cat. 76104, RNA Protect Tissue Reagent, Qiagen, Hilden, Germania) and stored at −80 °C pending extraction. All procedures were approved by the Scientific Committee of the University of Catania (code: 28_09_2020_CT) and performed in accordance with the Declaration of Helsinki as amended or comparable ethical standards.

### 2.2. RNA Extraction

Viral RNA is usually extracted from liquid and biological matrices (serum, saliva, plasma or swab buffer). In this study, substantial changes to current viral RNA extraction protocols were made in order to effectively obtain and increase viral RNA from decomposed lung specimens (Figure S1). Lung specimens were collected and transferred with disposable tweezers into 1.5 mL sterile tubes containing RNA Later as a stabilizer buffer. Frozen tissues were thawed on ice and then centrifuged at 6000× *g* for five minutes at 4 °C to eliminate the RNA Later buffer. Subsequently, tissues were placed in a 100 mm dish and a fragment of 4 × 4 mm was selected for RNA extraction.

**Figure 1:**
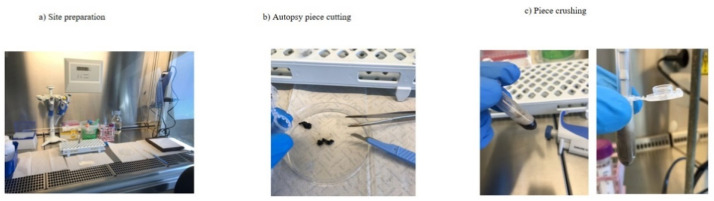
SARS-CoV 2 viral extraction from pulmonary tissue. (**a**) Preparation of the workplace, (**b**) pulmonary tissue cut for RNA extraction and (**c**) tissue destruction by T10 T10 ULTRA-TURRAX.

Samples were processed using a custom RNA extraction protocol combining two different commercial kits, i.e., QIAshredder (Cat. 79654, Qiagen, Hilden, Germany) and QIAamp Viral RNA (Cat. 52906, Qiagen, Hilden, Germany) for sample homogenization and total RNA extraction, respectively (Figure 1).

Some steps of the two commercial kits were modified as described below: 800 µL of AVL lysis buffer was supplemented with 8 µg of carrier RNA (Cat. 52906, QIAamp Viral RNA Mini Kit, Qiagen, Hilden, Germany); to promote sample lysis, a second round of homogenization was performed using the T10 ULTRA-TURRAX homogenizer (Cat. 0003737000, IKA^®^-Werke GmbH & Co. KG, Darmstadt, Germany) with disposable tips for volumes ranging from 500 to 2000 µL (mechanical disruption was performed by placing the tubes at room temperature and on ice every 30″(″=seconds) for two minutes in order to prevent sample warming and, consequently, RNA degradation); this phase lasted 10′ (′=minutes) in total, equivalent to the incubation time required by the AVL buffer. The lysed samples were transferred directly into QIAshredder columns to further homogenize tissues. Columns were centrifuged at full speed for 2′ and the flow through was collected in a new 1.5 mL tube for the subsequent steps of extraction. The QIAshredder lysate obtained was centrifuged at 1500× *g* for 10′ at room temperature in order to remove cell debris that could potentially interfere with the extraction procedure in the QIAamp Mini column. The supernatant was then transferred into a new collection tube and 800 µL ethanol (96–100%) was added and mixed by pulse-vertexing. The lysate (approximately 1600 µL) was applied into the QIAamp Mini column and centrifuged at 6000× *g* for 2 min, transferring no more than 630 µL of lysate for each centrifugation step and discarding the flow through. After lysate centrifugation, the column was washed with 350 µL AW1 washing buffer and centrifuged at 6000× *g* for 1′, discarding the flow through after centrifugation. Considering the minimal quantity of viral RNA compared to the total amount of nucleic acids extracted, an additional step with DNase I (Cat. 15200-40—Qiagen, Hilden, Germany) was performed in order to prevent the DNA from interfering with the extraction protocol (see technical notes). The column was treated with 80 μL of DNase I mix (10 μL DNasi and 70 μL Buffer RDD) and incubated at room temperature for 15′. After incubation, the column was washed again with 350 μL of AW1 washing buffer and centrifuged at 20,000× *g* for 1′. A third wash step was performed by applying 750 μL of AW2 washing buffer to the QIAamp Mini column, which was then centrifuged at 6000× *g* for 1′ with the lid open (after centrifugation, the QIAamp Mini column was placed into a new 2 mL collection tube and centrifuged at full speed for 1′ to dry the membrane completely). After washing, the QIAamp Mini column was transferred into a clean 1.5 mL tube. Then, 40 μL of AVE elution buffer were added to the column and incubated at room temperature for one minute. Finally, the RNA was eluted by centrifuging the column at 6000× *g* for 3′.

The extracted RNA was subsequently stored at −80°C.

The QIAamp Viral RNA Mini Kit protocol does not include DNase digestion; however, it was demonstrated that SARS-CoV-2 RNA is minimally represented compared to host DNA in different biological samples [12]. Therefore, in order to facilitate RNA extraction and avoid potential DNA contamination, DNase I was added during the first wash step with the AW1 chaotropic washing buffer containing surfactants useful in disrupting the interface between hydrophobic and hydrophilic molecules, thus facilitating DNA digestion.

### 2.3. RNA Quantification and Reverse Transcription

The custom extraction protocol here adopted allowed the isolation of total RNA from all samples included in the study. Total RNA concentration was determined by fluorometric assay. RNA quantity was tested by Qubit^®^ 3.0 Fluorometer (Cat. Q33216; Life Technologies; Thermo Fisher Scientific, Waltham, MA, USA) using the Qubit RNA HS Assay kit (250 pg/μL and 100 ng/μL). Reverse transcription was performed using 5 µL of total RNA and random primer hexamers (Cat. 18080-400, Superscript III; Thermo Fisher Scientific, Waltham, MA, USA). However, the amount of total RNA obtained for some samples was not sufficient for Qbit fluorescent quantification; to overcome this issue, relative fluorescent unit (RFU) quantification was used applying the following formula:

(1) $$x=\frac{RFu\;Sample\times 100}{RFu\;SD2}\;\left(e.g.,\;x=\frac{92.29\times 100}{168.22}=1.10\frac{ng}{\mu L}\right)$$

Due to the low amount of RNA obtained, a fixed volume of 5 µL of total RNA was used for cDNA synthesis (Table 1).

### 2.4. RT-qPCR and ddPCR Molecular Analyses

**Figure 2:**
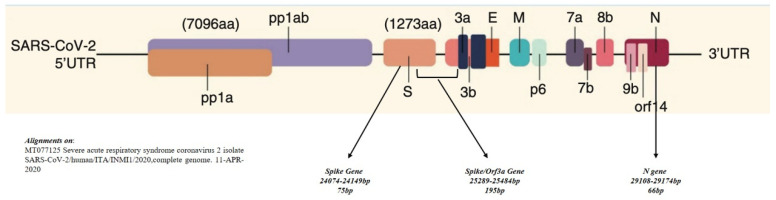
Gene structure of SARS-CoV-2. The position of the genes and primers amplified in this work were: *Spike* gene, from 24,074bp to 24,149 bp, amplicon size 75 pb; *Spike/Orf3a* gene, from 25,289 to 25,484 bp, amplicon size 195 bp; and *N* gene, from 29,108 to 29,174, amplicon size 66 bp.

The primers and probes used for RT-qPCR and droplet digital PCR (ddPCR) are reported in Table 2 and represented in Figure 2 (adapted from Zhang LP et al. [13]).

TaqMan RT-qPCR analyses were performed using the Quanti-Nova™ Probe PCR kit (Cat. 208252; Qiagen, Hilden, Germany) following the manufacturer’s procedure and at the following thermal conditions: PCR initial activation step at 95 °C for 2′; two step-cycling: denaturation at 95 °C for 5″, combined annealing/extension at 60 °C for 5″; for 45 cycles. PCR efficiency and expression rate were calculated using the Light Cycler^®^ 480 Software (Roche, Basel, Switzerland). Similarly, SYBR-Green RT-qPCR analysis was performed using the QuantiTect Syber-Green PCR kit (Cat. 204145; Qiagen, Hilden, Germany) following the manufacturer’s procedure and at the following thermal conditions: PCR initial activation step at 95 °C for 15′, three step-cycling: denaturation at 94 °C for 15″, annealing at 60 °C for 30″, extension at 72 °C for 30″, for 45 cycles. PCR efficiency and expression rate were calculated using the Light Cycler^®^ 480 Software (Roche, Basel, Switzerland). All reactions were run in duplicate. SARS-CoV-2 synthetic RNA (Cat. HE0060S, Helix Elite™ Synthetic Standard, Grenoble, France) was used as a positive control for RT-qPCR detection of the *N* gene. A Ct threshold of 35 was set to assess the positivity of samples. The cDNA obtained from tissue samples was further analyzed by droplet digital PCR (ddPCR) for the detection of SARS-CoV-2 nucleic acids and human RNA. The ddPCR mix was obtained and processed as previously described [12].

After amplification, negative and positive droplets were analyzed using the QX200 Droplet Reader and the QuantaSoft software (Bio-Rad v. 1.7.4, Pleasanton, CA, USA) as previously described [15,16]. All experiments were performed in duplicate. Finally, the 196 bp *Spike/Orfa3* gene junction fragment was amplified by end-point PCR to assess fragmentation of the extracted RNA.

### 2.5. End-Point PCR

cDNA was tested for all samples for a large third portion of SARS-CoV-2, the *Spike/Orfa3* gene junction. The primers used are reported in Table 2. End-point PCR was performed to detect the presence or absence of the amplified fragment. The PCRs were performed with Platinum Taq DNA Polymerase (Cat. 10966018, Invitrogen, Thermo Fisher, Monza, Italy) using 5 µL of cDNA. The program used was: initial denaturation 95 °C for 5′, 35 cycles of: annealing 60 °C for 1′, extension 72 °C for 1′, and denaturation 95 °C for 1′; final step 72 °C for 10′. The 196 bp amplicons obtained were verified on 18% agarose gel, in Tris Borate EDTA Buffer (TBE) (Cat. B52, Tris-borate-10X, Thermo Fisher, Monza, Italy), stained with SYBR™ Safe DNA Gel Stain (Cod. S33102, Invitrogen, Thermo Fisher, Monza, Italy) and 100 bp DNA Ladder (Cod. BR0800201, Biotechrabbit, Duesseldorf, Germany).

### 2.6. Sequencing

The three randomly selected amplified targets (N, Spike and Spike/Orfa3) obtained by end-point PCR were enzymatically purified with ExoSap (Cat. 78200, Applied Biosystem, Thermo Fisher, Monza, Italy) quantified by fluorimeter Qubit dsDNA BR Assay kit (Cat. 32850; Invitrogen; Thermo Fisher Scientific, Monza, Italy); 5 ng of the product was sequenced in a SeqStudio Genetic Analyzer (Thermo Fisher Scientific, Monza, Italy) using the Applied Biosystems BigDye terminator cycle sequencing 3.1v (Cat. 4337455; Thermo Fisher Scientific, Monza, Italy) as previously described [17]. The obtained sequence was compared with the reference sequence “MT077125 severe acute respiratory syndrome coronavirus 2 isolated SARS-CoV-2/human/ITA/INMI1/2020 (complete genome sequence release date: 11 April 2020)” using the BLAST tool (https://blast.ncbi.nlm.nih.gov/Blast.cgi).

### 2.7. Correlation Analysis

To establish a potential correlation among the length of burial, RNA input and expression of SARSCoV-2 *N* gene, *Spike* gene and *human glyceraldehyde 3-phosphate dehydrogenase* (*GAPDH*), Pearson’s correlation analyses were performed for the 16 long-buried patients taking into account both RT-qPCR and ddPCR results. This correlation matrix is a statistical tool that measures the linear correlation between two variables, X and Y. It has a value between +1 and −1, where +1 indicates total positive linear correlation, 0 no linear correlation, and −1 total negative linear correlation. The correlation coefficient ranges from −1 to 1, where 1 implies that a linear equation describes the relationship between X and Y perfectly, with all data points lying on a line for which Y increases as X increases [18].

### 2.8. Statistical Analyses

The QuantaSoft software (Bio-Rad v. 1.7.4, Pleasanton, CA, USA) was used for absolute quantification of the SARS-CoV-2 targets. Kolmogorov–Smirnov normality test was performed to assess the distribution of copies/µL of the targets analyzed. GraphPad Prism v.8 was used to perform Student’s t-test and Pearson’s correlation analyses.

## 3. Results

In this study, 18 corpses of patients who died from COVID-19 infection were exhumed to collect lung samples for the detection of SARS-CoV-2 viral RNA. Of these patients, 16 were exhumed after a long period of burial (ranging from 24 to 78 days), whereas autopsies were performed immediately after death (one to five days) in the other two patients, with corpses stored at a controlled temperature. Table S1 shows the sociodemographic and clinical characteristics of the patients enrolled in this study.

The custom protocol described was firstly applied to a fresh lung tissue sample obtained from a patient who died from a non-infectious disease, used as a control (Figure S2). The amount of RNA extracted for all samples is reported in Table 1.

### 3.1. RT-qPCR and ddPCR SARS-CoV-2 Detection

The samples included in this study were tested with three different chemistries and systems: RT-qPCR with Taq-Man probes and SYBR chemistry, ddPCR with TaqMan probes, and end-point PCR with Taq Platinum DNA polymerase. Results are reported in Table 3.

The detection of the SARS-CoV-2 *Spike* gene by SYBR Green RT-qPCR revealed that four out of the 16 decomposed tissue samples had no detectable amplification signals, while the remaining 12 samples showed an average threshold cycle (Ct) value of 25.6 ± 5.2 standard deviation (SD). As for the two fresh cadaveric tissues, the average Ct of the *Spike* gene was 31 ± 5.9 SD. Noteworthily, two decomposed tissues had very low Ct values of 15.52 and 19.37 for samples P9 and P10, respectively, suggesting the presence of a higher viral load. Also of note, one of the two fresh cadaveric tissues showed a late Ct of 35.2, suggesting a lower viral load compared to the other fresh tissue.

The use of RT-qPCR and specific TaqMan probes allowed detection of the SARS-CoV-2 *N* gene and human *GAPDH* in all analyzed samples; however, human RNA was only found in ten out of 16 decomposed tissue samples. In particular, *N* gene amplification signals were obtained from all decomposed tissues with an average Ct of 27.9 ± 4.0 SD. Similar results were obtained for fresh lung tissues, whose average Ct was 27.9 ± 1.9 SD. With regard to *GAPDH*, the ten samples with positive signals had an average Ct of 28.1 ± 1.97 SD, against 26.3 ± 3.25 SD of fresh samples. Therefore, no statistical differences were observed between decomposed and fresh cadaveric tissues in terms of Ct values.

Instead, absolute quantification of the human *GAPDH* and SARS-CoV-2 *N* gene by ddPCR showed major differences between decomposed and fresh cadaveric tissues that were not observed at RT-qPCR. As expected, *GAPDH* absolute quantification revealed better RNA quality in fresh cadaveric tissues compared to decomposed tissues, resulting in an average copies/μL value of 16.8 and 3, respectively (almost six-fold higher). Regarding SARS-CoV-2 *N* gene quantification, heterogeneous results were obtained in decomposed and fresh cadaveric tissues. On one hand, similar to RT-qPCR, this gene was detected in all samples analyzed regardless of sample degradation and length of burial. On the other, despite the long period of burial, some of the older tissues showed similar or even higher *N* gene levels (410.1090 and 50.600 copies/μL) compared to fresh tissues (122 and 621 copies/μL).

These data suggest that, despite degradation processes, human RNA and viral RNA can be still detected up to two months after death, especially in patients whose death is strictly related to severe COVID-19 pneumonia (e.g., P3, P9, P10, P14 and P15).

### 3.2. Correlation Analysis

All patients included in this work were exhumed after a period of time ranging from 24 to 78 days, with an average of 50.19 days ± 13.04 SD (Table 3). Pearson’s correlation analyses revealed differences in the behavior of variables. No correlation existed between the length of burial and amount of RNA obtained (*p*-value 0.099), as well as between length of burial and the human *GAPDH* recovered (*p*-value 0.529). Instead, a correlation was found between RNA input (in ng) and *GAPDH* expression, though at the limit of statistical significance (*p*-value 0.048). The SARS-CoV-2 *N* and *Spike* genes analyzed by both RT-qPCR and ddPCR showed no statistically valid correlation with the length of burial (*N*-gene *p*-value 0.744 and *Spike* gene *p*-value 0.783) (Figure 2 and Figure S3). On the contrary, there was a statistically significant correlation between total RNA input and detection of gene expression which was more marked for the Spike than for the *N* gene (*p*-values 0.001 vs. 0.069). The expression of viral genes was statistically significant compared to the human housekeeping *GAPDH* gene, with a *p*-value of 0.019 and 0.021 for the *N* and the *Spike* gene, respectively. These data suggest that the amount of total RNA and viral RNA was independent from the length of burial, and that the adopted extraction protocol was able to isolate RNA from degraded tissues. Noteworthily, taking into account the Ct values obtained for the SARS-CoV-2 genes, a strong, significant correlation was observed between the *N* and the *Spike* gene (Figure 2, *p*-value < 0.001). Therefore, both genes can be used for COVID-19 post-mortem confirmatory analyses.

Interestingly, while the data suggest that the amount of viral RNA was independent from tissue degradation, they still point to a link with viral load at the time of death, the latter being statistically related to the amount of total RNA extracted.

### 3.3. SARS-CoV-2 Sequencing

**Figure 3:**
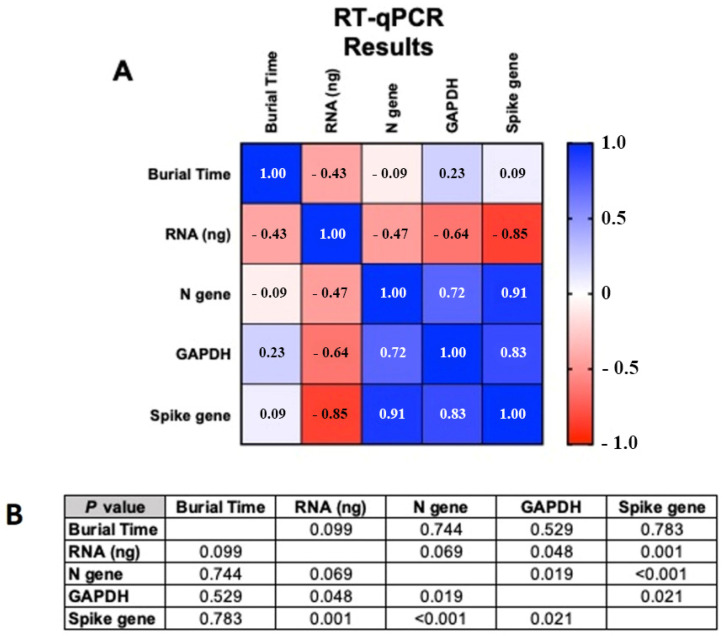
(**A**) Pearson’s correlation matrix between RT-qPCR cDNA, *GAPDH*, *N* e *Spike* viral genes and (**B**) *p*-value relative to the matrix, *p*-value <0.05.

As the end-point RT-PCRs failed to amplify the 195 bp Spike/Orfa3 fragment and conversely, we were able to amplify small *N* and *Spike* gene fragments (66 bp and 75 bp respectively), we have assumed the existence of a highly fragmented RNA filament (Figure 3). This further demonstrated the effectiveness of our extraction and amplification protocol in detecting SARS-CoV-2 viral RNA.

**Figure 4:**
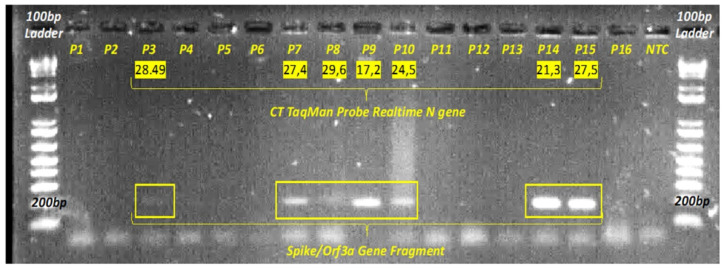
End-point PCR of the *Spike/Orf3a* gene fragment (198 bp). The images were underlined and correlated to the Ct obtained by TaqMan RTq-PCR of the *N* gene.

In order to avoid confounding results due to non-specific amplification signals, all targets were sequenced by the Sanger method. As reported in Figure 4, a perfect match was obtained between amplified fragments of the SARS-CoV-2 *N* gene, *Spike* gene and *Spike/Orfa3* and the SARS-CoV-2 reference sequence “MT077125 severe acute respiratory syndrome coronavirus 2 isolated SARS-CoV-2/human/ITA/INMI1/2020 (complete genome sequence release date: 11 April 2020)” (Figure S4).

## 4. Discussion and Conclusions

Since the SARS-CoV-2 outbreak was declared a pandemic by the WHO, the number of infections and related deaths has increased worldwide exponentially [19]. This has highlighted the importance of implementing shared guidelines for the management of COVID-19-positive and potentially infectious corpses [20,21]. Due to the huge amount of COVID-19 related-deaths, autopsies and confirmatory analyses could not be performed in all suspected cases immediately, therefore the scientific community has raised the question of whether confirmatory analyses could be performed on exhumed bodies after a long period of burial [22]. Moreover, the current knowledge about SARS-CoV-2 persistence in corpses is very poor. In fact, while previous studies have reported that MERS-CoV can be detected in nasal swabs up to three days after death [23], similar studies are scarce and often contradictory for SARS-CoV-2 [24].

In this scenario, autopsies could be performed both for clinical and forensic purposes. In the case of forensic investigations, post-mortem examinations could be performed even after a considerable post-mortem interval (PMI). Moreover, according to international procedures, bodies could be buried in a double-layer cloth sheet dipped in disinfectant and then packed in an additional double-layer sheet soaked with disinfectant (containing chlorine). Nevertheless, this study proved that it is possible to demonstrate the presence of SARS-CoV-2 at the time of death even if a considerable PMI is elapsed. Undoubtedly, these data are very useful for forensic purposes, demonstrating the presence of the virus in exhumed corpses. To the best of our knowledge, this study showed, for the first time, that SARS-CoV-2 RNA can be found in lungs after a long post-mortem interval (PMI) of up to 78 days and even in spite of appropriate burial practices. SARS-CoV-2 viral RNA detection is even more effective when using methods less susceptible to RNA quality, like ddPCR. Finally, both RT-qPCR and ddPCR proved effective in correctly detecting SARS-CoV-2 *N* gene expression, although ddPCR showed more robust results.

These innovative results further highlight the importance of forensic investigations even after a long PMI in order to confirm the presence of SARS-CoV-2 and ascertain the precise causes of death. This study also provides important insights into the procedures that should be adopted in the analysis of decomposed tissues and on how to overcome technical issues related to nucleic acid fragmentation.

The statistical data derived from the simple observation of Ct in qRT-PCR and of copies/µL in ddPCR showed a lack of correlation between length of burial and expression of all three genes evaluated (24 to a maximum of 78 days with an average of 50.19 days ± 13.04 SD) (Table 3). Instead, a correlation emerged with the initial RNA input (in ng), though not linear across the three genes. This may depend on differences in RNA conservation within the tissue, whereby the data seem to suggest less degradation for viral RNA compared to human RNA [25]. Furthermore, another difference emerges in the dosage between the *N* and *Spike* genes, with prevalence of expression for the *Spike* gene. This could be due to the complex mechanisms of viral replication creating different “accumulations” of viral nucleic acid tracts [26].

Despite the limitations of this study, mainly represented by the limited number of cases analyzed and the detection of SARS-CoV-2 RNA in lung tissue excluding other anatomical sites, our results strongly encourage COVID-19 post-mortem confirmatory analyses even after long periods of time after death. Noteworthily, our approach can only demonstrate the presence of COVID-19 infection at the time of death, however, the precise cause of death can only be assumed and presumably attributed to SARS-CoV-2. Nevertheless, the clinical data obtained from the individuals enrolled in this study revealed an almost significant alteration of platelet numbers, probably due to alternation of the coagulative cascade and platelet activation pathway related to COVID-19 infection, as demonstrated by other studies [27,28,29].

On these bases, further validation studies on a larger cohort of tissue samples are needed to confirm these preliminary results and clearly associate the presence of SARS-CoV-2 RNA with the actual cause of death of individuals who died with a suspicious COVID-19 infection.

## Figures and Tables

**Table 1:** Concentrations in ng/µL of RNA and total amount of cDNA per reaction per sample.

Sample	ng/µL	ng for cDNA Synthesis
P1	8.96	44.80
P2	0.88	4.40
P3	1.41	7.06
P4	1.47	7.36
P5	0.94	4.72
P6	0.93	4.63
P7	2.05	10.24
P8	2.19	10.96
P9	7.04	35.20
P10	6.24	31.20
P11	5.04	25.20
P12	2.25	11.26
P13	1.10	5.49
P14	1.52	7.60
P15	3.00	15.01
P16	0.34	1.68
P17	46.63	233
P18	43.96	220

**Table 2:** Primers and probes used for SARS-CoV-2 detection.

Gene	Primers	Oligonucleotide Sequence (5′→3′)	Label	Working Concentration	Amplicon Size bp	Used for	References
**N**	2019-nCov_N-F	TTACAAACATTGGCCGCAAA	None	20 μM		End-Point PCR	CDC, 2020 [10]
	2019-nCov_N-R	GCGCGACATTCCGAAGAA	None	20 μM	66	RT-qPCR	
	2019-nCov_N-P	FAM-ACAATTTGCCCCCAGCGCTTCAG -BHQ1	FAM, BHQ-1	5 μM		ddPCR	
**Spike**	2019-nCov_Spike-F	CGGCCTTACTGTTTTGCCAC	None	20 μM	75 bp	Syber Green	Falzone et al., 2020 [12]
	2019-nCov_Spike-R	TGTACCCGCTAACAGTGCAG	None	20 μM		RT-qPCR	
**Spike/Orfa3**	2019-nCov_Spike/Orfa3-F	TGAGCCAGTGCTCAAAGGAG	None	20 μM	195	End-Point PCR	this paper
	2019-nCov_Spike/Orfa3-R	CGCCAACAATAAGCCATCCG	None	20 μM			
**GAPDH**	hGAPDH-F	CATGAGAAGTATGACAACAGCC	None	20 μM	115	RT-qPCR	Carpenter et al., 2005 [14]
	hGAPDH-R	TGAGTCCTTCCACGATACC	None	20 μM		ddPCR	
	hGAPDH-P	FAM- AGCAATGCCTCCTGCACCACCAA -BHQ1	FAM, BHQ-1	5 μM			

The table reports the oligonucleotide sequence for *N* gene, *Spike* gene, *Spike/Orfa3* and Human Control gene *GAPDH*, label, working concentration, amplicon size and technique used for each gene amplified. Primers for Spike/Orfa3 were designed and analyzed with the online software: http://www.premierbiosoft.com/netprimer/. F, forward primers; R, reverse primers; P, probe.

**Table 3:** Human *GAPDH* and SARS-CoV-2 *N*, *Spike* genes expression by RT-qPCR and ddPCR and burial days and dates of death and autopsy of the 16 long-buried COVID-19 patients.

Sample ID	TaqMan RT-qPCR	ddPCR Absolute Quantification	TaqMan RT-qPCR	ddPCR Absolute Quantification	Spike Gene			
	GAPDH	GAPDH	N Gene	N Gene	SYBR RT-qPCR	Death Date	Autopsy Date	Burial Days
	(*C*t Value)	(Copies/μL Reaction)	(*C*t Value)	(Copies/μL Reaction)	(*C*t Value)			
**Decomposed** **Tissues**								
P1	26.6	6.0	28.9	10.2	ND	10/05/2020	11/06/2020	32
P2	ND	3.9	29.9	3.1	ND	02/05/2020	16/06/2020	45
P3	ND	2.3	28.5	160	28.98	20/04/2020	17/06/2020	58
P4	ND	3.0	30.8	9.6	29.1	03/05/2020	11/06/2020	39
P5	ND	3.1	29	6.4	30.1	18/04/2020	18/06/2020	61
P6	ND	2.8	29.2	5	30.9	15/04/2020	18/06/2020	64
P7	ND	3.1	27.4	169	25.9	29/04/2020	18/06/2020	50
P8	27.5	3.3	29.6	4.5	30.9	01/05/2020	16/06/2020	46
P9	25.4	2.5	17.2	50600	15.5	22/04/2020	17/06/2020	56
P10	26.4	3.0	24.5	410	19.4	24/04/2020	17/06/2020	54
P11	28.6	2.4	28.2	1.1	24.9	04/05/2020	11/06/2020	38
P12	31.6	2.5	29.8	1	ND	26/04/2020	16/06/2020	51
P13	28.9	1.9	30.2	3.2	ND	25/04/2020	16/06/2020	52
P14	26.6	3.5	21.3	1090	22.2	23/04/2020	17/06/2020	55
P15	28.5	3.0	27.5	135	28.2	18/05/2020	11/06/2020	24
P16	30.7	2.4	30.2	4.7	31.3	01/04/2020	18/06/2020	78
**Fresh** **Cadaveric** **Tissue**								
P17	28.6	19.5	26.5	621	26.8			
P18	24.0	14.1	29.3	12.2	35.2			
**Decomposed vs. Fresh Cadaveric** **Tissue**								
*p*-value	0.578	0.121	0.888	0.363	0.2699			

## Data Availability

The data presented in this study are available in supplementary material.

## Supplementary Materials

The following are available online at https://www.mdpi.com/article/10.3390/diagnostics11071158/s1, Table S1. Socio-demographic and clinical-pathological features of COVID-19 patients. Figure S1. SARS-CoV 2 viral extraction from pulmonary tissues workflow. Figure S2. (A) qPCR on non-infected samples. (B) qPCR performed to assure the validity of the extraction method and the absence of cross-contamination. Figure S3. (A) Pearson’s correlation matrix between the cDNA amount and the ddPCR results obtained for *GAPDH*, *N* and *Spike* viral genes and (B) *p*-value relative to the matrix, *p*-value ≤0.05. Figure S4. Electropherograms of SARS-CoV-2 fragments. (A) SARS-CoV-2 *N* gene sequence; (B) SARS-CoV-2 *Spike* gene sequence; SARS-CoV-2 c) *Spike/Orf3a* gene sequence. *N* and *Spike* sequences were aligned with the reference genome MW041156, while the *Spike/Orf3a* sequence was aligned with the reference genome MT077125.
