# Supplementary material for: Post-Mortem Detection of SARS-CoV-2 RNA in Long-Buried Lung Samples
Source: Diagnostics (Basel). 2021 Jun 24;11(7):1158. doi: 10.3390/diagnostics11071158 (PMC8304625; doi:10.3390/diagnostics11071158)

Supplementary material

Table S1. Socio-demographic and clinical-pathological features of COVID-19 patients

	Clinical Findings	
	Old Tissues (16 Samples)	Fresh Tissues (2 Samples)
**Age (years)**	83.5	75.5
**Sex**	8M, 8F	2F
Male (%)	8 (50%)	0 (0%)
Female (%)	8 (50%)	2 (100%)
**Comorbidities**		
Hypertension	11 (68.75%)	2 (100%)
Diabetes	5 (31.25%)	1 (50%)
Chronic pulmunary diseases	2 (12.5%)	---
Obesity (BMI > 30)	3 (18.75%)	---
Cardiovascular diseases	11 (68.75%)	---
Chronic renal diseases	3 (18.75%)	---
Cancer	3 (18.75%)	---
Alzheimer's disease	1 (6.25%)	---
**Burial time (days)**	50.19 (24 – 78)	3

	Laboratory Findings (CI 95%)		
	First exams after COVID-19 positivity	Last exams before death	*P*-value
**Redcells (x 10^6/μL)**	4.49 (4.04 - 4.94)	4.12 (3.66 - 4.58)	0,219
**Hemoglobin (g/dL)**	12.64 (11.28 - 14.00)	11.75 (10.46 - 13.05)	0,303
**Hematocrit (%)**	38.85 (34.72 - 42.98)	36.17 (32.19 - 40.16)	0,308
**Platelets (10^3/μL)**	254.3 (175.49 - 333.11)	160.45 (96.37 - 224.52)	0,052
**White Blood Cells (10^3/μL)**	18.8 (2.98 - 40.57)	12.1 (6.8 - 17.34)	0,513
**Neutrofils**	8.2 (3.68 - 12.74)	10.8 (5.31 - 16.33)	0,415
**Lymphocytes**	0.99 (0.55 - 1.42)	0.82 (0.38 - 1.26)	0,549
**Neutrofils/Lymphocyte Ratio**	8,28	13,17	NA
**C-reactive protein (mg/L)**	159.5 (56.75 - 262.3)	84.71 (17.44 - 186.9)	0,258
**Prothrombin time (sec)**	12.96 (9.04 - 16.87)	18.46 (6.89 - 30.02)	0,308
**Prothrombin activation (%)**	81.56 (61.87 - 101.2)	72.71 (41.85 - 103.6)	0,573
**International Normalized Ratio (INR)**	1.23 (0.98 - 1.48)	1.6 (0.59 - 2.62)	0,419
**Partial thromboplastin time (sec)**	28.79 (26.45 - 31.13)	37.86 (25.12 - 50.60)	0,135
**D-dimer (ng/mL)**	754.1 (309.4 - 1199)	845.7 (474.5 - 1217)	0,711
**Erythrocyte Sedimentation Rate (ESR) (mm/h)**	92.4 (50.21 - 134.6)	56 (29.44 - 141.4)	0,214
**Fibrinogen (mg/dL)**	595.9 (421.5 - 770.2)	435.9 (291 - 580.8)	0,12
**LDH (UI/L)**	911.9 (561.3 - 1262)	963.9 (385.9 - 1542)	0,859

Figure S1. SARS-CoV 2 viral extraction from pulmonary tissues work flow**.**

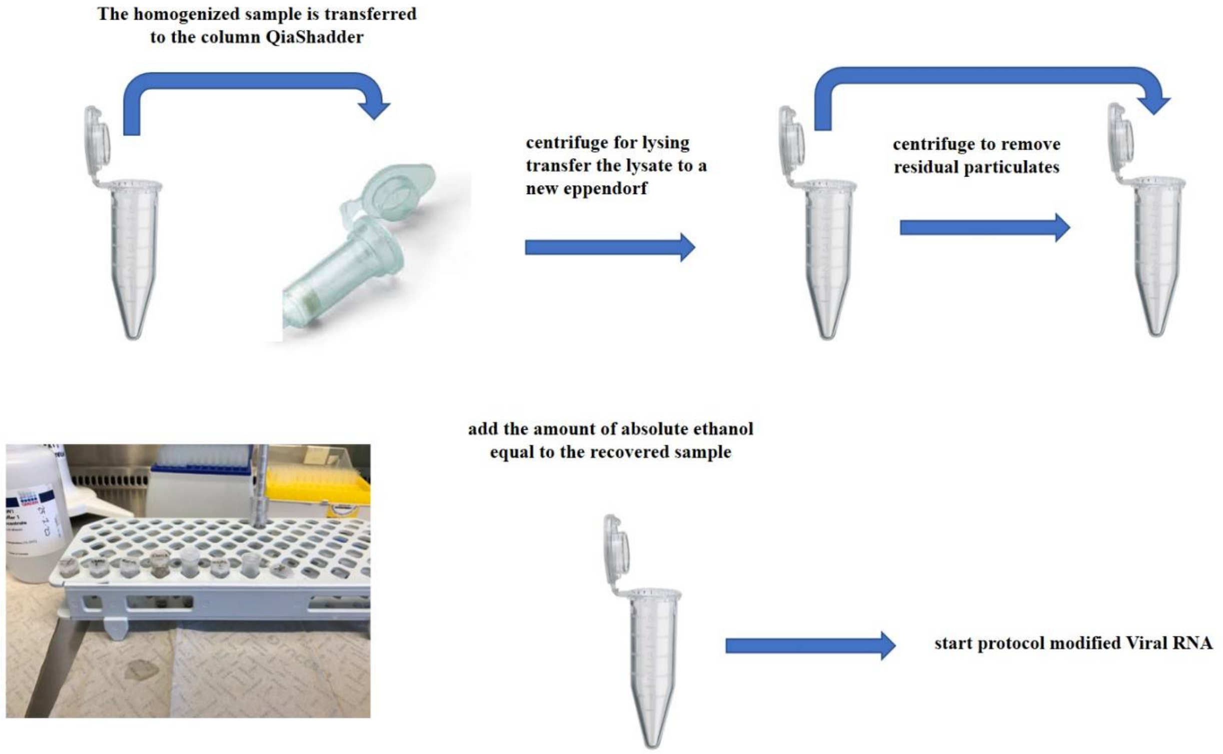

Figure S2. A) RT-qPCR detection of SARS-CoV-2 N2 gene in non-infected samples. B) RT-qPCR detection of human GAPDH in control samples and lung tissues to assess the validity of the extraction protocol**.**

A)

	Name	Ct
A	SARS-CoV2 N2 Lung Method 1	NA
B	SARS-CoV2 N2 Lung Method 1	NA
C	SARS-CoV2 N2 Primers CTRL Pos	20.21
D	SARS-CoV2 N2 Primers CTRL Pos	18,29
E	SARS-CoV2 N2 NTC	NA

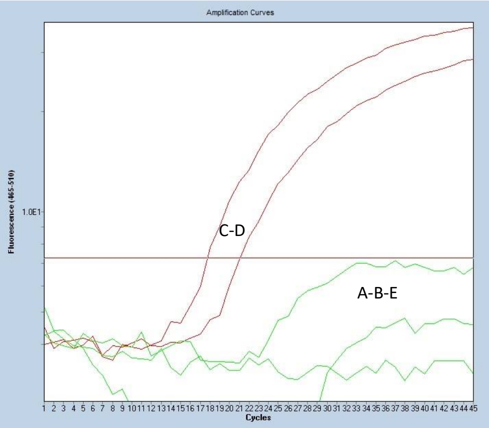

Legend: A-B) Analysis of SARS-CoV-2 N2 gene expression in normal lung (performed in duplicate); C-D) Analysis of SARS-CoV-2 N2 genes in a positive control obtained for a patient with a confirmed diagnosis of COVID-19 infection (performed in duplicate); E) Non-Template Control.

B)

	Name	Ct
A	GAPDH Negative Sample 1	NA
B	GAPDH Negative Sample 1	NA
C	GAPDH Lung Method 2	34.80
D	GAPDH Lung Method 2	36.86
G	GAPDH Lung Method 1	27.98
H	GAPDH Lung Method 1	26.67
I	GAPDH MG-63 Human Cell Line	17.46
F	GAPDH NTC	NA

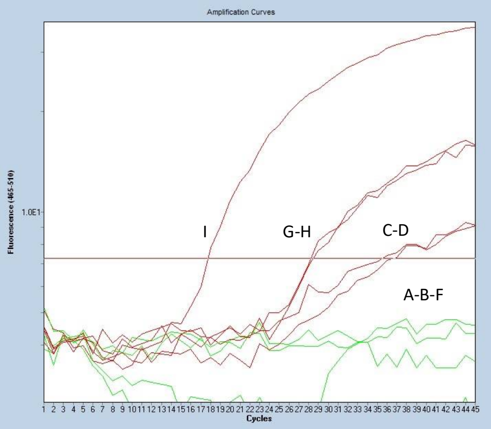

Legend: A-B) Negative samples consisting of all the buffers and components used during the extractions, tested to verify any possible cross-contamination. C-D-G-H) RNA extracted from a lung specimen obtained using two different

methods **(i.e. samples kept at room temperature or in ice)** and obtained from a patient negative to COVID-19 infection.

Figure S3. A) Pearson's correlation matrix between the cDNA amount and the ddPCR results obtained for GAPDH and SARS-CoV-2 N2 gene, from red to blue the increasing statistical significance and B) *p*-value relative to the matrix, *p*-value ≤0.05.

A

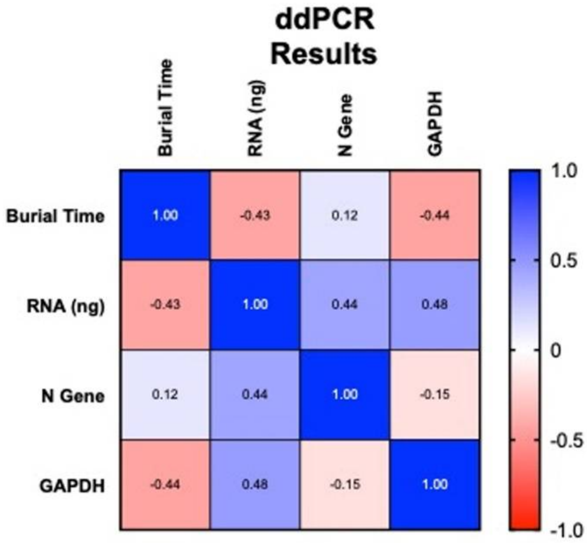

B

P value	Burial Time	RNA (ng)	N Gene	GAPDH
Burial Time	/	0.099	0.656	0.090
RNA (ng)	0.099	/	0.091	0.062
N Gene	0.656	0.091	/	0.571
GAPDH	0.090	0.062	0.571	/

Figure S4. Electropherograms of SARS-CoV-2 fragments. A) SARS-CoV-2 N gene sequence; B) SARS-CoV-2 Spike gene sequence; SARS-CoV-2 c) Spike/Orf3a gene sequence. N and Spike sequences were aligned with the reference genome MW041156, while the Spike/Orf3a sequence was aligned with the reference genome MT077125.

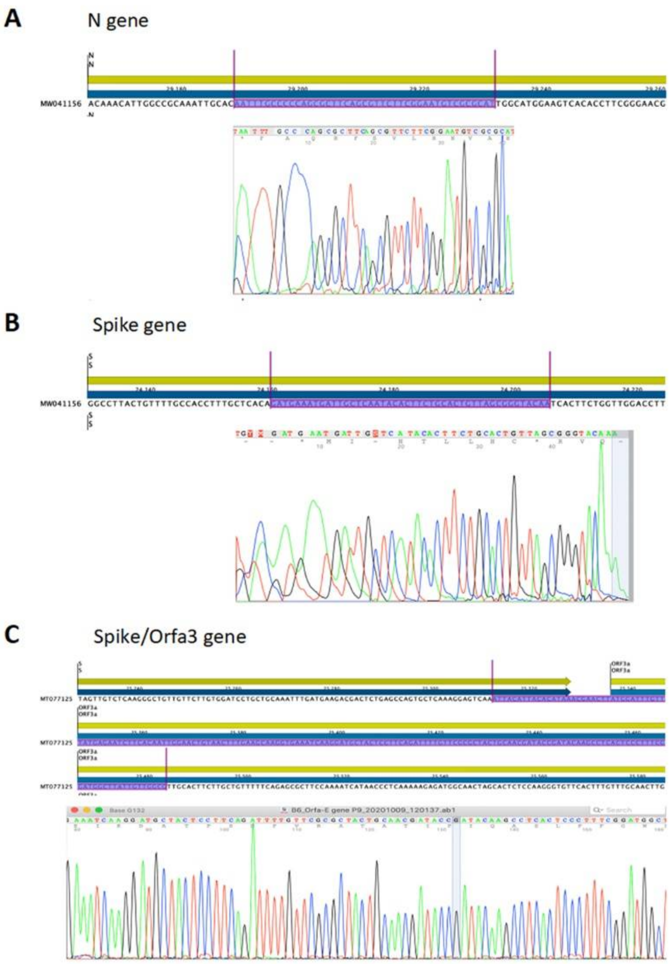

Supplement: Supplementary file 1 [file diagnostics-11-01158-s001.zip › diagnostics-1237453-supplementary.pdf]
